# Supplementary material for: Active regulation of the epidermal growth factor receptor by the membrane bilayer
Source: bioRxiv. 2025 Aug 18:2025.08.14.670284. Preprint. [Version 1] doi: 10.1101/2025.08.14.670284 (PMC12393268; doi:10.1101/2025.08.14.670284)
Supplement: Supplement 1 [file NIHPP2025.08.14.670284v1-supplement-1.pdf]

bioRxiv preprint doi: https://doi.org/10.1101/2025.08.14.670284; this version posted August 18, 2025. The copyright holder for this preprint (which was not certified by peer review) is the author/funder, who has granted bioRxiv a license to display the preprint in perpetuity. It is made available under aCC-BY 4.0 International license.

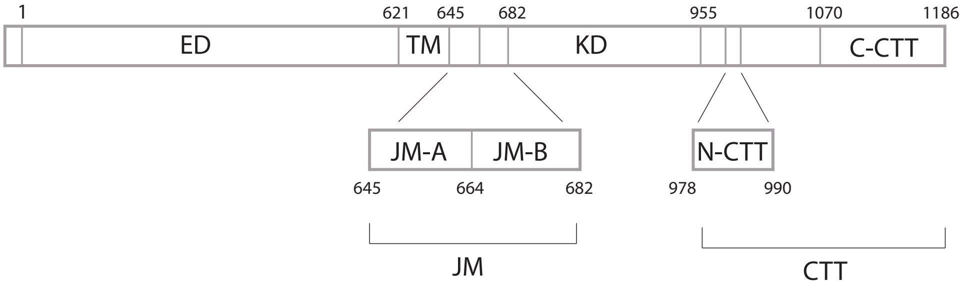

Supplementary Fig. 1. Domains of EGFR. EGFR consists of a 621-amino acid extracellular region (ED), a 24-amino acid transmembrane-spanning domain (TM), and an intracellular region, which is a 37-amino acid juxtamembrane domain (JM), a 273-amino acid kinase domain (KD) and a 231-amino acid C-terminal tail (CTT). The JM is further divided into juxtamembrane-A (JM-A) and juxtamembrane-B (JM-B) domains. Residues 978–990 are defined as N-terminal portion of the CTT (N-CTT) and residues 1070–1186 are defined as the C-terminal portion of the CTT (C-CTT). Residue numbering corresponds to EGFR excluding the signal sequence.

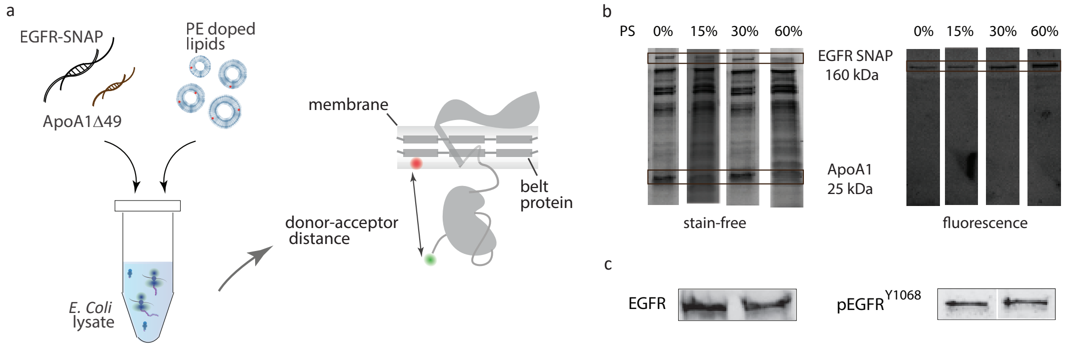

Supplementary Fig. 2. Production and characterization of full-length EGFR in nanodiscs. (a) Cell-free reaction for the production of EGFR and nanodisc belt protein. Codon optimized DNA of the receptor protein (EGFR-SNAP) and the belt protein (ApoA1Δ49) are incubated overnight together with lipid vesicles with or without cholesterol and $E.\ Coli$ lysate at 25° C. (b) Stain-free (left) and fluorescence (right) gel images of the His-tag purified sample show the presence of ApoA1 at 25 kDa and full-length EGFR at 160 kDa, which implies successful EGFR production and insertion into nanodiscs. The presence of the EGFR band alone in the fluorescence gel image indicates successful and specific labeling. (c) Western blots were performed on labelled EGFR in nanodiscs. Anti-EGFR Western blots (left) and anti-phosphotyrosine Western blots (right) tested the presence of EGFR and its ability to undergo tyrosine phosphorylation, respectively, consistent with previous experiments on similar preparations.[18,54,55]

bioRxiv preprint doi: https://doi.org/10.1101/2025.08.14.670284; this version posted August 18, 2025. The copyright holder for this preprint (which was not certified by peer review) is the author/funder, who has granted bioRxiv a license to display the preprint in perpetuity. It is made available under aCC-BY 4.0 International license.

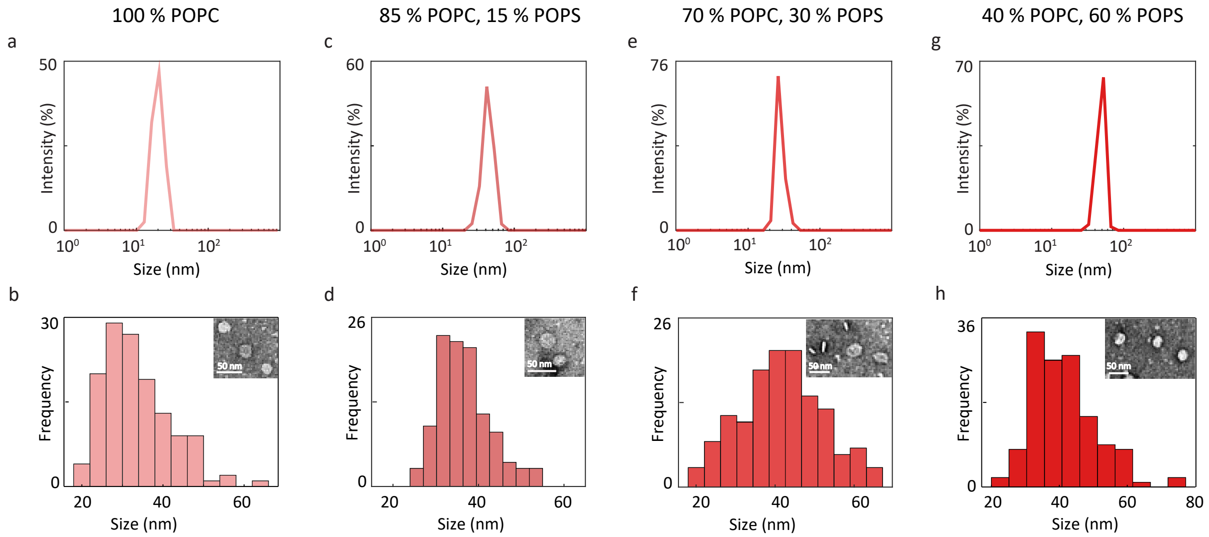

Supplementary Fig. 3. Characterization of EGFR-containing nanodiscs in different anionic membrane environments. (a) Dynamic light scattering (DLS) of EGFR in 100% POPC nanodiscs in PBS buffer indicates ∼38 nm average size. (b) Size distribution of EGFR in 100% POPC nanodiscs from negative stain transmission electron microscopy (TEM). The circles in the images (inset) show formation of 100% POPC nanodiscs. The mean disc diameter is 33.4 ± 8.5 nm (N = 134). (c) DLS of EGFR in 85% POPC, 15% POPS nanodiscs in PBS buffer indicates ∼40.7 nm average size. (d) Size distribution of EGFR in 85% POPC, 15% POPS nanodiscs from negative stain TEM. The circles in the images (inset) show formation of 85 % POPC, 15 % POPS nanodiscs. The mean disc diameter is 36.8 ± 6.0 nm (N = 116). (e) DLS of EGFR in 70% POPC, 30% POPS nanodiscs in PBS buffer indicates ∼54.8 nm average size. (f) Size distribution of EGFR in 70 % POPC, 30 % POPS nanodiscs from negative stain TEM. The circles in the images (inset) show formation of 70 % POPC, 30 % POPS nanodiscs. The mean disc diameter is 37.8 ± 9.7 nm (N = 223). (g) DLS of EGFR in 40 % POPC, 60 % POPS nanodiscs in PBS buffer indicates ∼46.2 nm average size. (h) Size distribution of EGFR in 40 % POPC, 60 % POPS nanodiscs from negative stain TEM. The circles in the images (inset) show formation of 40 % POPC, 60 % POPS nanodiscs. The mean disc diameter is 41.2 ± 9.7 nm (N = 133).

bioRxiv preprint doi: https://doi.org/10.1101/2025.08.14.670284; this version posted August 18, 2025. The copyright holder for this preprint (which was not certified by peer review) is the author/funder, who has granted bioRxiv a license to display the preprint in perpetuity. It is made available under aCC-BY 4.0 International license.

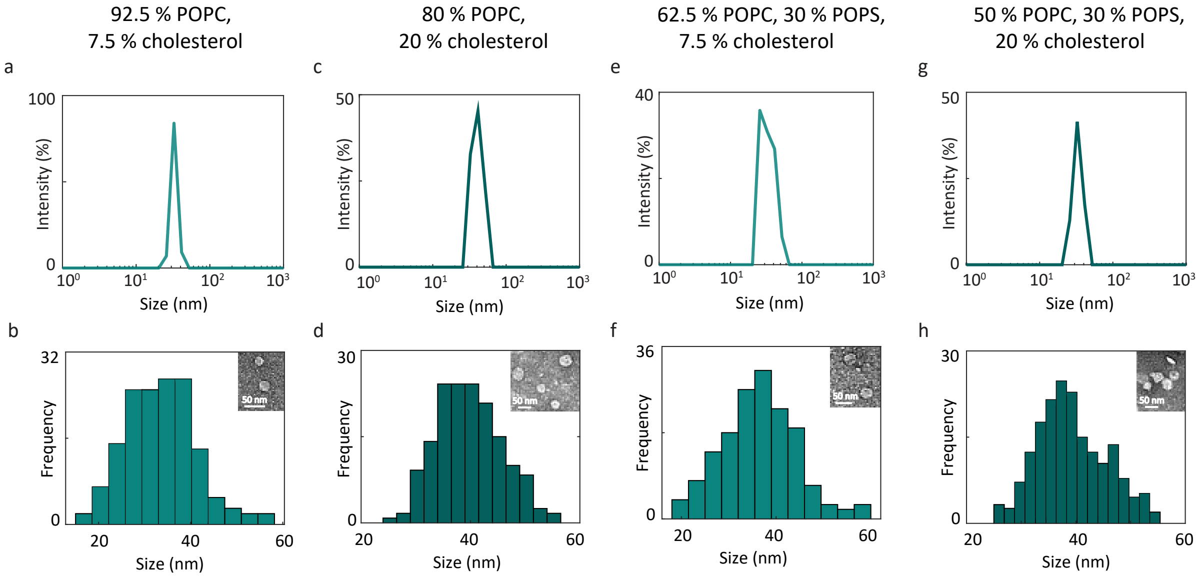

Supplementary Fig. 4. Characterization of EGFR-containing nanodiscs in membrane environments containing cholesterol. (a) Dynamic light scattering (DLS) of EGFR in 80% POPC, 20% cholesterol containing nanodiscs in PBS buffer indicates 39.6 nm average size. (b) Size distribution of EGFR in 80% POPC, 20% cholesterol containing nanodiscs from negative stain transmission electron microscopy (TEM). The circles in the images (inset) show formation of 80% POPC, 20% cholesterol nanodiscs. The mean disc diameter is 39.8 $\pm$ 5.9 nm (N = 188). (c) DLS of EGFR in 50% POPC, 30% POPC, 20% cholesterol nanodiscs in PBS buffer indicates 39.2 nm average size. (d) Size distribution of EGFR in 50% POPC, 30% POPC, 20% cholesterol nanodiscs from negative stain TEM. The circles in the images (inset) show formation of 50% POPC, 30% POPC, 20% cholesterol nanodiscs. The mean disc diameter is 39.2 $\pm$ 6.3 nm (N = 281). (e) DLS of EGFR in 92.5% POPC, 7.5% cholesterol nanodiscs in PBS buffer indicates 32.8 nm average size. (f) Size distribution of EGFR in 92.5% POPC, 7.5% cholesterol nanodiscs from negative stain TEM. The circles in the images (inset) show formation of 92.5% POPC, 7.5% cholesterol nanodiscs. The mean disc diameter is 33.6 $\pm$ 7.7 nm (N = 154). (g) DLS of EGFR in EGFR in 62.5% POPC, 30% POPS, 7.5% cholesterol nanodiscs in PBS buffer indicates 33 nm average size. (h) Size distribution of EGFR in 62.5% POPC, 30% POPS, 7.5% cholesterol nanodiscs from negative stain TEM. The circles in the images (inset) show formation of 62.5% POPC, 30% POPS, 7.5% cholesterol nanodiscs. The mean disc diameter is 36.5$\pm$ 7.8 nm (N = 159).

bioRxiv preprint doi: https://doi.org/10.1101/2025.08.14.670284; this version posted August 18, 2025. The copyright holder for this preprint (which was not certified by peer review) is the author/funder, who has granted bioRxiv a license to display the preprint in perpetuity. It is made available under aCC-BY 4.0 International license.

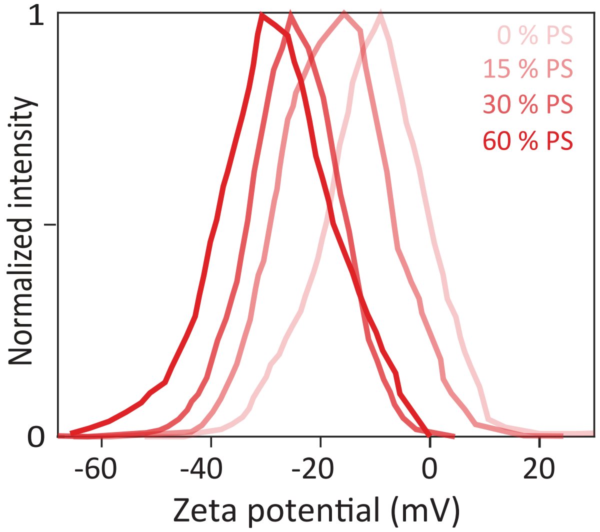

Supplementary Fig. 5. Characterization of anionic content in EGFR-embedded nanodiscs with zeta potential.[56] Zeta potential distributions for EGFR in nanodiscs containing increasing amounts of anionic lipids (0%, 15%, 30% and 60% POPS). Maximum values from the distributions are shown in Fig. 1b in the main text. Error bars are from three technical replicates.

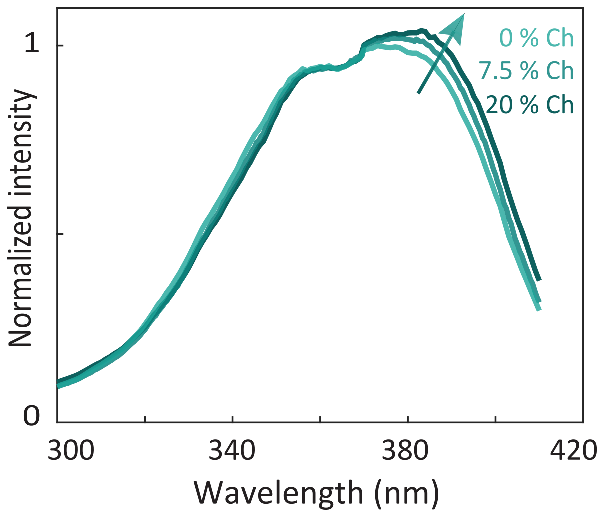

Supplementary Fig. 6. Characterization of cholesterol (Ch) content in EGFR-embedded nanodiscs with Laurdan. Ensemble fluorescence excitation spectra ($\lambda_{em} = 440$ nm) of EGFR embedded Laurdan containing nanodiscs with 0% cholesterol, 7.5% cholesterol and 20% cholesterol. In the Laurdan excitation spectra, increase in the excitation band centered around 390 nm is observed with the addition of increasing amounts of cholesterol to the EGFR embedded nanodisc.[57]

bioRxiv preprint doi: https://doi.org/10.1101/2025.08.14.670284; this version posted August 18, 2025. The copyright holder for this preprint (which was not certified by peer review) is the author/funder, who has granted bioRxiv a license to display the preprint in perpetuity. It is made available under aCC-BY 4.0 International license.

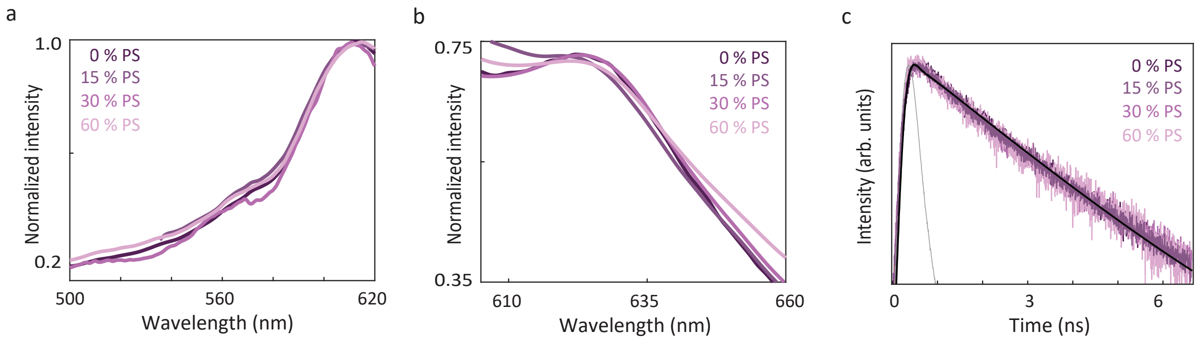

Supplementary Fig. 7. Characterization of ss594 labeled EGFR nanodiscs in different anionic lipid environments. (a) Ensemble fluorescence excitation spectra ($\lambda_{em} = 660$ nm) of ss594 labeled EGFR nanodiscs with 0% POPS, 15% POPS, 30% POPS and 60% POPS lipids. (b) Ensemble fluorescence emission spectra ($\lambda_{exc} = 565$ nm) of ss594 labeled EGFR nanodiscs in 0% POPS, 15% POPS, 30% POPS and 60% POPS lipids. (c) Ensemble time-correlated single photon counting measurements for ss594 labeled EGFR nanodiscs in 0% POPS, 15% POPS, 30% POPS and 60% POPS lipids. The instrument response function (IRF) is shown in gray.

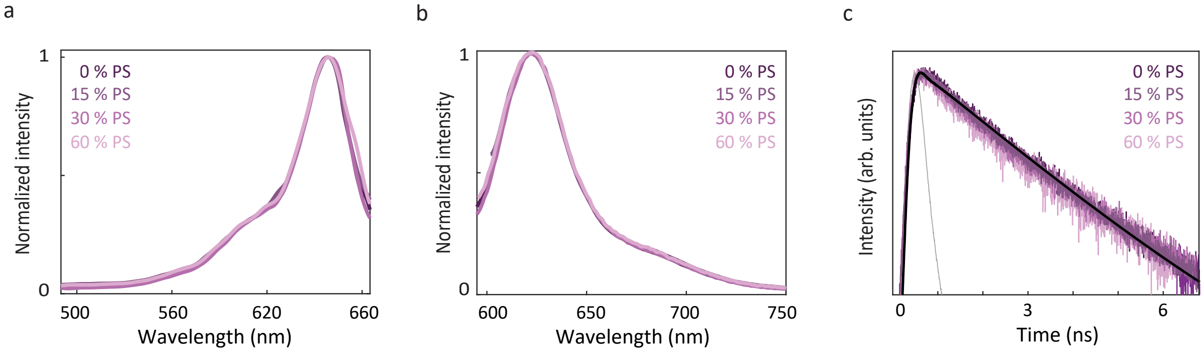

Supplementary Fig. 8. Characterization of cy5 labeled EGFR nanodiscs in different anionic lipid environments. (a) Ensemble fluorescence excitation spectra ($\lambda_{em} = 700$ nm) of cy5 labeled nanodiscs in 0% POPS, 15% POPS, 30% POPS and 60% POPS lipids. (b) Ensemble fluorescence emission spectra ($\lambda_{ex} = 630$ nm) of cy5 labeled nanodiscs in 0% POPS, 15% POPS, 30% POPS and 60% POPS lipids. (c) Ensemble time-correlated single photon counting measurements were performed for cy5 labeled nanodiscs in 0% POPS, 15% POPS, 30% POPS and 60% POPS. The instrument response function (IRF) is shown in gray.

bioRxiv preprint doi: https://doi.org/10.1101/2025.08.14.670284; this version posted August 18, 2025. The copyright holder for this preprint (which was not certified by peer review) is the author/funder, who has granted bioRxiv a license to display the preprint in perpetuity. It is made available under aCC-BY 4.0 International license.

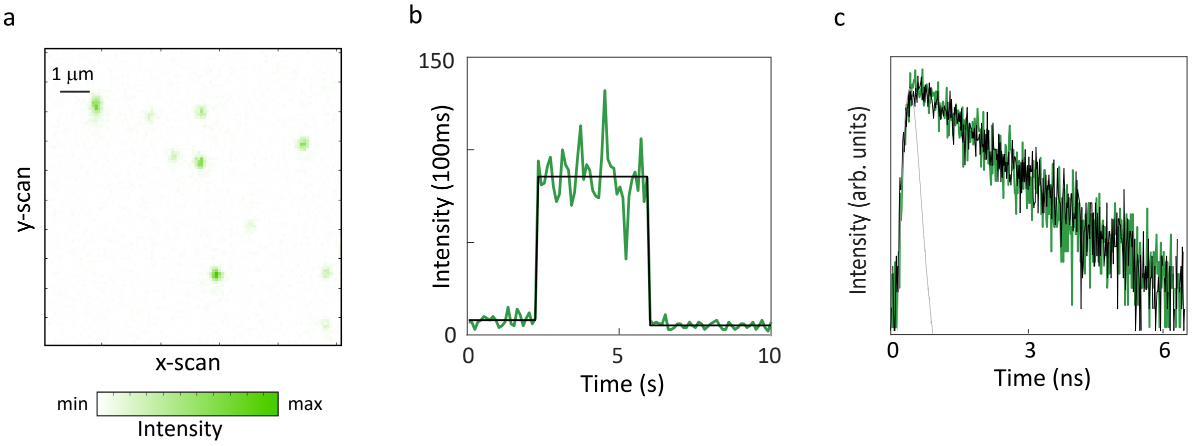

Supplementary Fig. 9. Single ss594 labeled EGFR embedded nanodiscs. (a) Confocal fluorescence image of immobilized constructs of EGFR in nanodiscs labeled with ss594 ($\lambda_{exc} = 550$ nm). (b) Representative intensity time trace from a single construct. The number of detected photons for each 100 ms interval was calculated and used to generate a fluorescence intensity trace (green) with the average intensity for the emissive period overlaid (black). (c) Histogram of the arrival times of detected photons generates the donor lifetime decay profile. Representative decay profiles of EGFR (green) with fit curve (black). The instrument response function (IRF) is shown in gray.

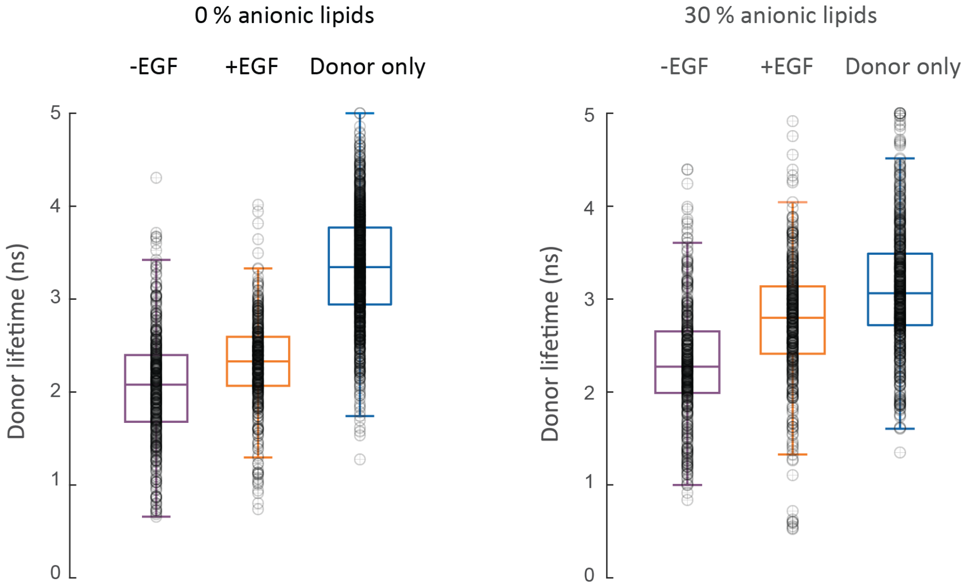

Supplementary Fig. 10. Box plots of the donor lifetime distributions from smFRET experiments to measure the distance between the ATP binding site and the C-terminus of EGFR. Distributions of the donor lifetime from the histograms in Fig. 2e, f of main text are represented as box plots along with the donor-only samples. One-way ANOVA was performed to obtain the P-values (Supplementary Table 2). The median value of the donor only distribution in each lipid environment was used as the reference value for calculations of the donor-acceptor distances for all smFRET measurements in that environment.

bioRxiv preprint doi: https://doi.org/10.1101/2025.08.14.670284; this version posted August 18, 2025. The copyright holder for this preprint (which was not certified by peer review) is the author/funder, who has granted bioRxiv a license to display the preprint in perpetuity. It is made available under aCC-BY 4.0 International license.

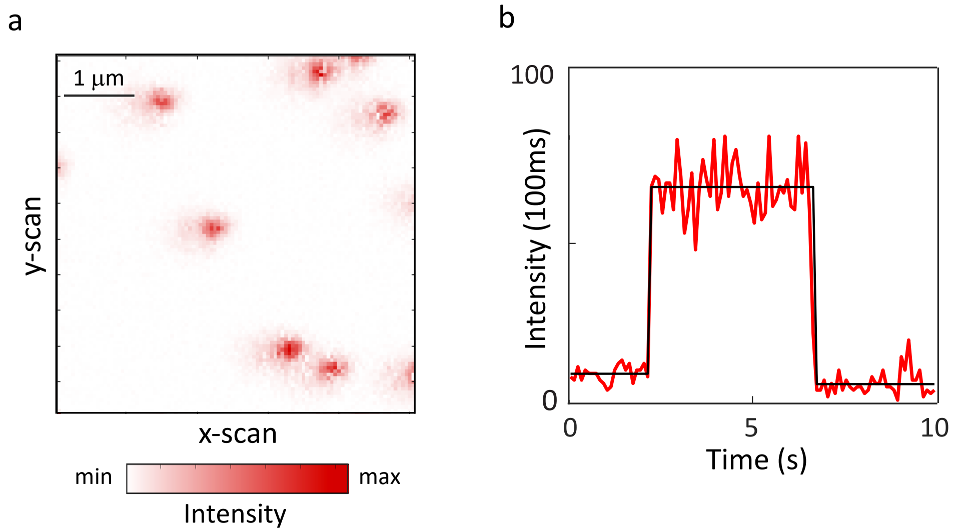

Supplementary Fig. 11. Single Cy5 labeled EGFR embedded nanodiscs. (a) Confocal fluorescence image of immobilized constructs of EGFR in nanodiscs containing a labeled Cy5 lipid ($\lambda_{exc} = 640$ nm). (b) Representative intensity time trace from a single construct. The number of detected photons for each 100 ms interval was calculated and used to generate a fluorescence intensity trace (red) with the average intensity for the emissive period overlaid (black).

bioRxiv preprint doi: https://doi.org/10.1101/2025.08.14.670284; this version posted August 18, 2025. The copyright holder for this preprint (which was not certified by peer review) is the author/funder, who has granted bioRxiv a license to display the preprint in perpetuity. It is made available under aCC-BY 4.0 International license.

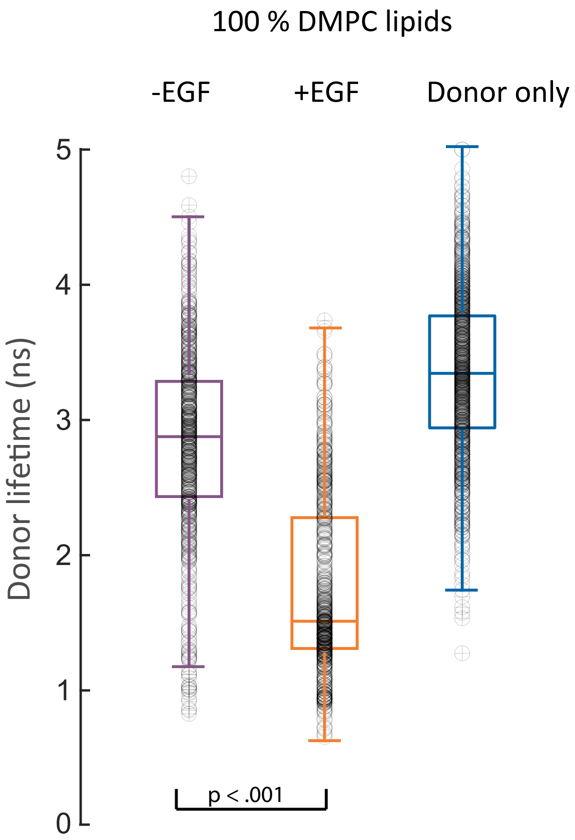

Supplementary Fig. 12. Box plots of the donor lifetime distributions in 100% DMPC lipid environment. Distributions of the donor lifetime from the histograms in Fig. 3b of main text are represented as box plots along with the donor-only sample. One-way ANOVA was performed to obtain the P-values (Supplementary Table 5). The median value of the donor only distribution in each lipid environment was used as the reference value for calculations of the donor-acceptor distances for all smFRET measurements in that environment.

bioRxiv preprint doi: https://doi.org/10.1101/2025.08.14.670284; this version posted August 18, 2025. The copyright holder for this preprint (which was not certified by peer review) is the author/funder, who has granted bioRxiv a license to display the preprint in perpetuity. It is made available under aCC-BY 4.0 International license.

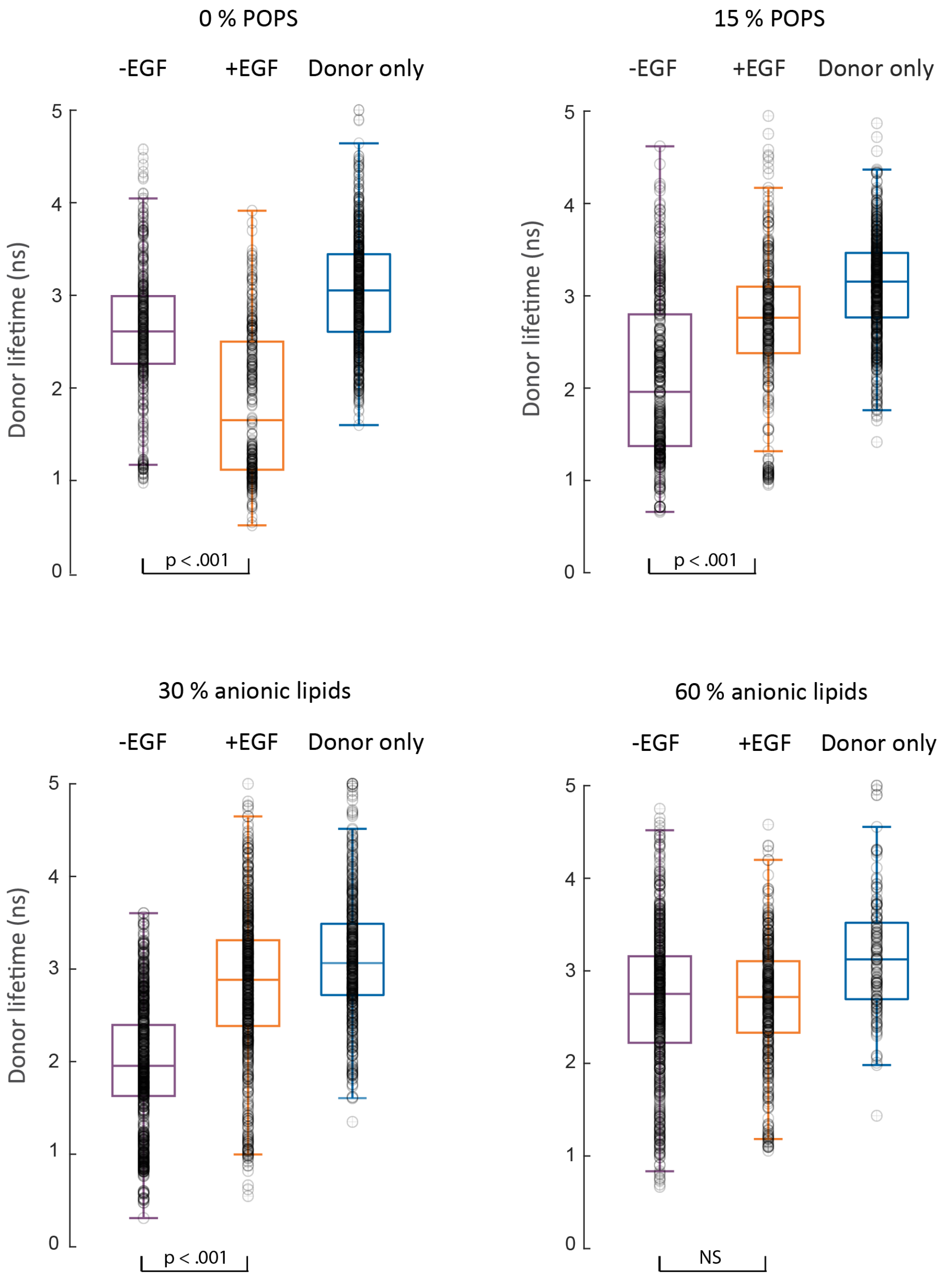

Supplementary Fig. 13. Box plots of the donor lifetime distributions in different anionic lipid environments. Distributions of the donor lifetime from the histograms in Fig. 3c of main text are represented as box plots along with the donor-only sample. One-way ANOVA was performed to obtain the P-values (Supplementary Table 5). The median value of the donor only distribution in each lipid environment was used as the reference value for calculations of the donor-acceptor distances for all smFRET measurements in that environment.

bioRxiv preprint doi: https://doi.org/10.1101/2025.08.14.670284; this version posted August 18, 2025. The copyright holder for this preprint (which was not certified by peer review) is the author/funder, who has granted bioRxiv a license to display the preprint in perpetuity. It is made available under aCC-BY 4.0 International license.

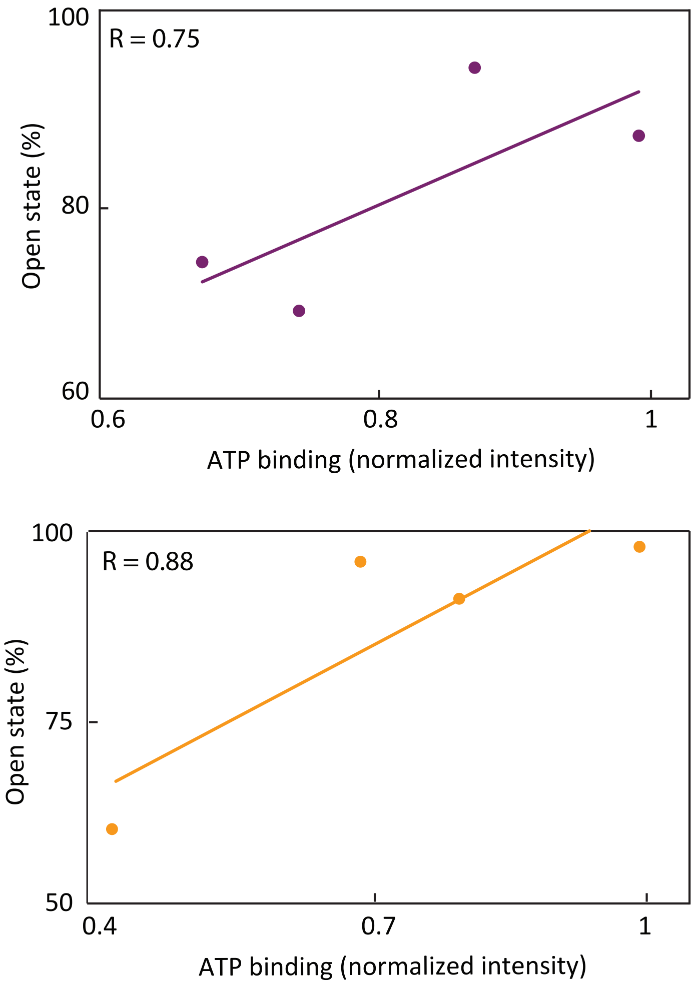

Supplementary Fig. 14. EGFR intracellular domain conformation is correlated with ATP binding. (a) Correlation analysis between distance of EGFR C-terminus from the bilayer and extent of ATP binding in the (top) absence of EGF (Pearson correlation R = 0.75) and (bottom) presence of EGF (Pearson correlation R = 0.88).

bioRxiv preprint doi: https://doi.org/10.1101/2025.08.14.670284; this version posted August 18, 2025. The copyright holder for this preprint (which was not certified by peer review) is the author/funder, who has granted bioRxiv a license to display the preprint in perpetuity. It is made available under aCC-BY 4.0 International license.

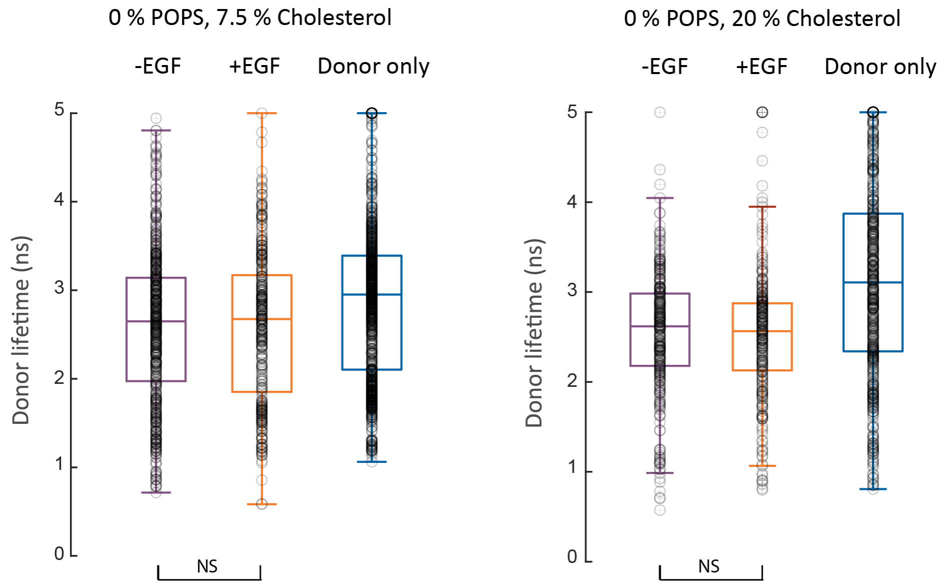

Supplementary Fig. 15. Box plots of the donor lifetime distributions in different cholesterol environments. Distributions of the donor lifetime from the histograms in Fig. 3e of main text are represented as box plots along with the donor-only sample. One-way ANOVA was performed to obtain the P-values (Supplementary Table 9). The median value of the donor only distribution in each lipid environment was used as the reference value for calculations of the donor-acceptor distances for all smFRET measurements in that environment.

bioRxiv preprint doi: https://doi.org/10.1101/2025.08.14.670284; this version posted August 18, 2025. The copyright holder for this preprint (which was not certified by peer review) is the author/funder, who has granted bioRxiv a license to display the preprint in perpetuity. It is made available under aCC-BY 4.0 International license.

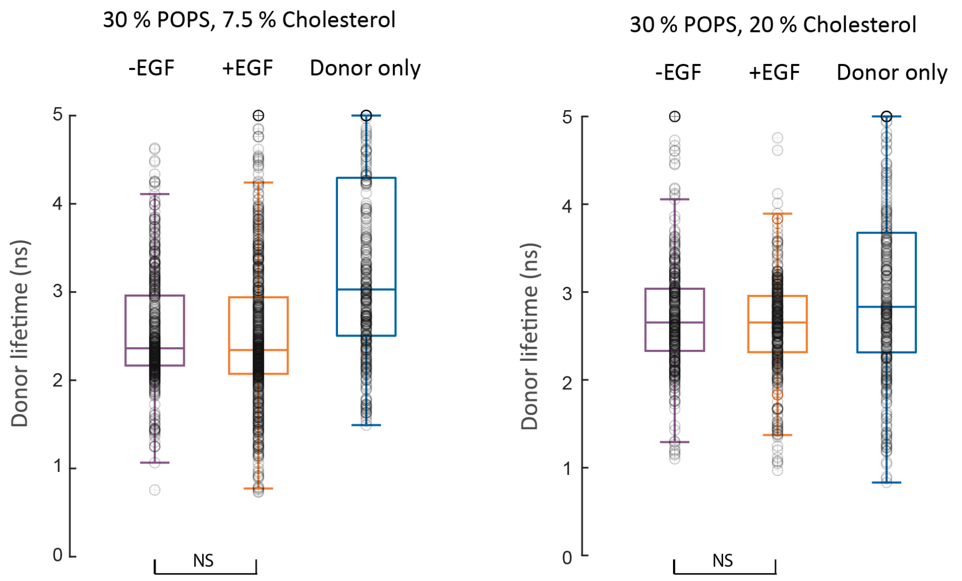

Supplementary Fig. 16. Box plots of the donor lifetime distributions in different lipid environments. Distributions of the donor lifetime from the histograms in Fig. 3g of main text are represented as box plots along with the donor-only sample. One-way ANOVA was performed to obtain the P-values (Supplementary Table 13). The median value of the donor only distribution in each lipid environment was used as the reference value for calculations of the donor-acceptor distances for all smFRET measurements in that environment.

bioRxiv preprint doi: https://doi.org/10.1101/2025.08.14.670284; this version posted August 18, 2025. The copyright holder for this preprint (which was not certified by peer review) is the author/funder, who has granted bioRxiv a license to display the preprint in perpetuity. It is made available under aCC-BY 4.0 International license.

Experiments	Experiment distance (nm)	Simulation distance (nm)
0 % anionic lipids		
EGFR, -EGF	8.15 [8.00, 8.24]	7.00 [6.00, 8.00]
EGFR, +EGF	8.62 [8.50, 8.75]	5.84 [5.17, 7.17]
30 % anionic lipids		
EGFR, -EGF	9.06 [8.93, 9.20]	5.66 [5.66, 5.66]
EGFR, +EGF	11.64 [11.11, 12.56]	7.50 [6.17, 9.17]

Supplementary Table 1. Median distances between the ATP binding site (residue 721) and the C-terminal end of the protein from smFRET experiments and simulations. The distance values were extracted as the medians of the distributions in Figs. 2e-h in the main text which show that the simulations replicate the experimental trends. The numbers in parenthesis indicates the 95 % confidence interval for experiments and the minimal and maximal median value from three equal partitions of data for simulations. Ro = 7.5 nm for snap surface 594 and atto 647N

Experiment 1	Experiment 2	P-value	F	Degrees of freedom
0 % anionic lipids				
EGFR, -EGF	EGFR, +EGF	< 0.001	49.4	875
30 % anionic lipids				
EGFR, -EGF	EGFR, +EGF	< 0.001	108.8	863

Supplementary Table 2. Statistical analysis of smFRET lifetime distributions. Results from one-way analysis of variance (ANOVA) with P-value, F-statistic and degrees of freedom for all experimental pairs (experiment 1 and experiment 2 in the above table) from the distributions reported in Fig. 2e, f in the main text.

bioRxiv preprint doi: https://doi.org/10.1101/2025.08.14.670284; this version posted August 18, 2025. The copyright holder for this preprint (which was not certified by peer review) is the author/funder, who has granted bioRxiv a license to display the preprint in perpetuity. It is made available under aCC-BY 4.0 International license.

Experiments	Number of molecules	Number of bunches
0 % anionic lipids		
EGFR, -EGF	111	448
EGFR, +EGF	92	428
30 % anionic lipids		
EGFR, -EGF	95	422
EGFR, +EGF	111	442

Supplementary Table 3. Sample sizes for smFRET measurements. The number of molecules and number of photon bunches used to construct the lifetime distributions are reported for all smFRET histograms from Fig. 2e, f in the main text.

bioRxiv preprint doi: https://doi.org/10.1101/2025.08.14.670284; this version posted August 18, 2025. The copyright holder for this preprint (which was not certified by peer review) is the author/funder, who has granted bioRxiv a license to display the preprint in perpetuity. It is made available under aCC-BY 4.0 International license.

Experiments	Distance (nm)
0 % POPS, 100 % DMPC; -EGF	11.39 [11.14, 11.65]
0 % POPS, 100 % DMPC; +EGF	8.14 [8.10, 8.22]
0 % POPS, 100 % POPC; -EGF	12.12 [11.65, 12.75]
0 % POPS, 100 % POPC; +EGF	8.80 [8.21, 9.59]
15 % POPS, 85 % POPC; -EGF	9.19 [8.80, 9.58]
15 % POPS, 85 % POPC; +EGF	11.92 [11.44, 12.40]
30 % POPS, 70 % POPC; -EGF	9.18 [9.08, 9.33]
30 % POPS, 70 % POPC; +EGF	*13.94 [12.50, 14.44]
60 % POPS, 40 % POPC; -EGF	11.36 [11.07, 11.73]
60 % POPS, 40 % POPC; +EGF	11.22 [10.94, 11.58]

Supplementary Table 4. Median distances between the membrane and C-terminal end of the protein from smFRET experiments. The distance values were extracted from the distributions shown in Fig. 3b, c in the main text. The numbers in parenthesis indicates the 95 % confidence interval for experiments. Asterisk (*) indicates distance was beyond the FRET range for the snap surface 594 and cy5 dye pair (Ro = 8.4 nm).[61]

Experiment 1	Experiment 2	P-value	F	Degrees of freedom
0 % POPS, 100 % DMPC; -EGF	0 % POPS, 100 % DMPC; +EGF	< 0.001	911.3	1398
0 % POPS, 100 % POPC; -EGF	0 % POPS, 100 % POPC; +EGF	< 0.001	204.48	757
15 % POPS, 85 % POPC; -EGF	15 % POPS, 85 % POPC; +EGF	< 0.001	104.87	850
30 % POPS, 70 % POPC; -EGF	30 % POPS, 70 % POPC; +EGF	< 0.001	540.13	1686
60 % POPS, 40 % POPC; -EGF	60 % POPS, 40 % POPC; +EGF	0.7512	0.1	1193

Supplementary Table 5. Statistical analysis of smFRET lifetime distributions. Results from one-way analysis of variance (ANOVA) with P-value, F-statistic and degrees of freedom for all experimental pairs (experiment 1 and experiment 2 in the above table) from the distributions reported in Fig. 3b, c in the main text.

bioRxiv preprint doi: https://doi.org/10.1101/2025.08.14.670284; this version posted August 18, 2025. The copyright holder for this preprint (which was not certified by peer review) is the author/funder, who has granted bioRxiv a license to display the preprint in perpetuity. It is made available under aCC-BY 4.0 International license.

Experiments	Number of molecules	Number of bunches
0 % POPS, 100 % DMPC; -EGF	51	702
0 % POPS, 100 % DMPC; +EGF	53	697
0 % POPS, 100 % DMPC; donor-only	77	992
0 % POPS, 100 % POPC; -EGF	74	422
0 % POPS, 100 % POPC; +EGF	75	336
0 % POPS, 100 % POPC; donor-only	122	1254
15 % POPS, 85 % POPC; -EGF	87	490
15 % POPS, 85 % POPC; +EGF	76	361
15 % POPS, 85 % POPC; donor-only	107	665
30 % POPS, 70 % POPC; -EGF	155	891
30 % POPS, 70 % POPC; +EGF	118	796
30 % POPS, 70 % POPC; donor-only	59	551
60 % POPS, 40 % POPC; -EGF	150	723
60 % POPS, 40 % POPC; +EGF	71	471
60 % POPS, 40 % POPC; donor-only	113	488

Supplementary Table 6. Sample sizes for smFRET measurements. The number of molecules and number of photon bunches used to construct the lifetime distributions are reported for all smFRET histograms from Fig. 3b, c in the main text.

bioRxiv preprint doi: https://doi.org/10.1101/2025.08.14.670284; this version posted August 18, 2025. The copyright holder for this preprint (which was not certified by peer review) is the author/funder, who has granted bioRxiv a license to display the preprint in perpetuity. It is made available under aCC-BY 4.0 International license.

Experiments	Compact state	Open state
0 % POPS, 100 % DMPC; -EGF	5.4 % [3.8 %, 6.9 %]	95 % [93 %, 96 %]
0 % POPS, 100 % DMPC; +EGF	58 % [54 %, 62 %]	42 % [38 %, 46 %]
0 % POPS, 100 % POPC; -EGF	13 % [10 %, 16 %]	87 % [84 %, 90 %]
0 % POPS, 100 % POPC; +EGF	40 % [34 %, 46 %]	60 % [54 %, 66 %]
15 % POPS, 85 % POPC; -EGF	31 % [25%, 37 %]	69 % [63 %, 75 %]
15 % POPS, 85 % POPC; +EGF	8.8 % [8.7 %, 9.0 %]	91 % [91 %, 91 %]
30 % POPS, 70 % POPC; -EGF	26 % [19 %, 33 %]	74 % [67 %, 81 %]
30 % POPS, 70 % POPC; +EGF	4.0 % [3.4 %, 4.6 %]	96 % [95 %, 97 %]
60 % POPS, 40 % POPC; -EGF	6.3 % [5.2 %, 7.4 %]	94 % [93 %, 95 %]
60 % POPS, 40 % POPC; +EGF	2.1 % [1.2 %, 2.9 %]	98 % [97 %, 99 %]

Supplementary Table 7. Amplitude of compact and open state of the EGFR intracellular domain from smFRET lifetime distributions in main text Fig. 3b, c. The numbers in parenthesis indicates the error bar indicated in Fig. 3h.

bioRxiv preprint doi: https://doi.org/10.1101/2025.08.14.670284; this version posted August 18, 2025. The copyright holder for this preprint (which was not certified by peer review) is the author/funder, who has granted bioRxiv a license to display the preprint in perpetuity. It is made available under aCC-BY 4.0 International license.

Experiments	Experiment distance (nm)
92.5 % POPC, 7.5 % Cholesterol; -EGF	12.08 [11.61, 12.65]
92.5 % POPC, 7.5 % Cholesterol; +EGF	12.23 [11.50, 13.24]
80 % POPC, 20 % Cholesterol; -EGF	11.12 [10.68, 11.47]
80 % POPC, 20 % Cholesterol; +EGF	10.87 [10.56, 11.25]

Supplementary Table 8. Median distances between the membrane and C-terminal end of the protein from smFRET experiments. The distance values were extracted from the distributions shown in Fig. 3e in the main text. The numbers in parenthesis indicates the 95 % confidence interval for experiments. Ro = 8.4 nm for snap surface 594 and cy5.[61]

Experiment 1	Experiment 2	P-value	F	Degrees of freedom
92.5 % POPC, 7.5 % Cholesterol; -EGF	92.5 % POPC, 7.5 % Cholesterol; +EGF	0.91	0.01	975
80 % POPC, 20 % Cholesterol; -EGF	80 % POPC, 20 % Cholesterol; +EGF	0.8245	0.05	600

Supplementary Table 9. Statistical analysis of smFRET lifetime distributions. Results from one-way analysis of variance (ANOVA) with P-value, F-statistic and degrees of freedom for all experimental pairs (experiment 1 and experiment 2 in the above table) from the distributions reported in Fig. 3e in the main text.

Experiments	Number of molecules	Number of bunches
92.5 % POPC, 7.5 % Cholesterol; -EGF	71	596
92.5 % POPC, 7.5 % Cholesterol; +EGF	59	380
92.5 % POPC, 7.5 % Cholesterol; donor-only	58	1255
80 % POPC, 20 % Cholesterol; -EGF	62	315
80 % POPC, 20 % Cholesterol; +EGF	58	286
80 % POPC, 20 % Cholesterol; donor-only	48	601

Supplementary Table 10. Sample sizes for smFRET measurements. The number of molecules and number of photon bunches used to construct the lifetime distributions are reported for all smFRET histograms from Fig. 3e in the main text.

bioRxiv preprint doi: https://doi.org/10.1101/2025.08.14.670284; this version posted August 18, 2025. The copyright holder for this preprint (which was not certified by peer review) is the author/funder, who has granted bioRxiv a license to display the preprint in perpetuity. It is made available under aCC-BY 4.0 International license.

Ligands	Compact state	Open state
92.5 % POPC, 7.5 % Cholesterol; -EGF	11 % [8.7 %, 13 %]	89 % [87 %, 91 %]
92.5 % POPC, 7.5 % Cholesterol; +EGF	10 % [7.2 %, 12 %]	90 % [88 %, 93 %]
80 % POPC, 20 % Cholesterol; -EGF	4.6 % [3.9 %, 5.4 %]	95 % [95 %, 96 %]
80 % POPC, 20 % Cholesterol; +EGF	6.0 % [5.1 %, 6.9 %]	94 % [93 %, 95 %]

Supplementary Table 11. Amplitude of compact and open state of the EGFR intracellular domain from smFRET lifetime distributions in main text Fig. 3e. The numbers in parenthesis indicates the error bar indicated in Fig. 3h.

bioRxiv preprint doi: https://doi.org/10.1101/2025.08.14.670284; this version posted August 18, 2025. The copyright holder for this preprint (which was not certified by peer review) is the author/funder, who has granted bioRxiv a license to display the preprint in perpetuity. It is made available under aCC-BY 4.0 International license.

Experiments	Experiment distance (nm)
62.5 % POPC, 30 % POPS, 7.5 % Cholesterol, -EGF	10.36 [10.24, 10.49]
62.5 % POPC, 30 % POPS, 7.5 % Cholesterol, +EGF	10.30 [10.19, 10.43]
50 % POPC, 30 % POPS, 20 % Cholesterol, -EGF	*12.95 [11.48, 12.27]
50 % POPC, 30 % POPS, 20 % Cholesterol, +EGF	*12.96 [11.48, 12.11]

Supplementary Table 12. Median distances between the membrane and C-terminal end of the protein from smFRET experiments. The distance values were extracted from the distributions shown in Fig. 3g in the main text. The numbers in parenthesis indicates the 95 % confidence interval for experiments. Asterisk (*) indicates distance was beyond the FRET range for the snap surface 594 and cy5 dye pair (Ro = 8.4m).[61]

Experiment 1	Experiment 2	P-value	F	Degrees of freedom
62.5 % POPC, 30 % POPS, 7.5 % Cholesterol, -EGF	62.5 % POPC, 30 % POPS, 7.5 % Cholesterol, +EGF	0.61	0.27	1203
50 % POPC, 30 % POPS, 20 % Cholesterol, -EGF	50 % POPC, 30 % POPS, 20 % Cholesterol, +EGF	0.002	9.88	794

Supplementary Table 13. Statistical analysis of smFRET lifetime distributions. Results from one-way analysis of variance (ANOVA) with P-value, F-statistic and degrees of freedom for all experimental pairs (experiment 1 and experiment 2 in the above table) from the distributions reported in Fig. 3g in the main text.

bioRxiv preprint doi: https://doi.org/10.1101/2025.08.14.670284; this version posted August 18, 2025. The copyright holder for this preprint (which was not certified by peer review) is the author/funder, who has granted bioRxiv a license to display the preprint in perpetuity. It is made available under aCC-BY 4.0 International license.

Experiments	Number of molecules	Number of bunches
62.5 % POPC, 30 % POPS, 7.5 % Cholesterol; -EGF	42	489
62.5 % POPC, 30 % POPS, 7.5 % Cholesterol; +EGF	55	715
62.5 % POPC, 30 % POPS, 7.5 % Cholesterol; donor-only	75	783
50 % POPC, 30 % POPS, 20 % Cholesterol; -EGF	81	390
50 % POPC, 30 % POPS, 20 % Cholesterol; +EGF	71	405
50 % POPC, 30 % POPS, 20 % Cholesterol; donor-only	66	877

Supplementary Table 14. Sample sizes for smFRET measurements. The number of molecules and number of photon bunches used to construct the lifetime distributions are reported for all smFRET histograms from Fig. 3g in the main text.

bioRxiv preprint doi: https://doi.org/10.1101/2025.08.14.670284; this version posted August 18, 2025. The copyright holder for this preprint (which was not certified by peer review) is the author/funder, who has granted bioRxiv a license to display the preprint in perpetuity. It is made available under aCC-BY 4.0 International license.

Ligands	Compact state	Open state
62.5 % POPC, 30 % POPS, 7.5 % Cholesterol, -EGF	0.0 % [0.0 %, 0.0 %]	100 % [100 %, 100 %]
62.5 % POPC, 30 % POPS, 7.5 % Cholesterol, +EGF	5.9 % [4.8 %, 7.0 %]	94 % [93 %, 95 %]
50 % POPC, 30 % POPS, 20 % Cholesterol, -EGF	0 % [0 %, 0 %]	100 % [100 %, 100 %]
50 % POPC, 30 % POPS, 20 % Cholesterol, +EGF	0.9 % [0.8 %, 1.0 %]	99 % [99 %, 99 %]

Supplementary Table 15. Amplitude of compact and open state of the EGFR intracellular domain from smFRET lifetime distributions in main text Fig. 3g. The numbers in parenthesis indicates the error bar indicated in Fig. 3h.

Antibody name	Target	Host	Company	Clone	Dilution
Anti-EGFR Antibody (A-10)	EGFR C-terminal	MouseIgG_{2a}	Santa Cruz Biotechnology	Monoclonal	1:200
Human Phospho-EGFR Y1068 Antibody	EGFR phosphorylated at Y1068	MouseIgG_{2a}	R&D Systems	Monoclonal	1:200
Goat anti-Mouse IgG (H+L) Highly Cross-Adsorbed Antibody Alexa Fluor 790	Mouse	GoatIgG	Thermo Fisher	Polyclonal	1:10000

Supplementary Table 16. List of antibodies used to show phosphorylation of EGFR nanodiscs.
